# Computed Tomographic Features in a Case of Bilateral Neoplastic Cryptorchidism with Suspected Torsion in a Dog

**DOI:** 10.3389/fvets.2016.00033

**Published:** 2016-04-27

**Authors:** Scott Stokowski, Jeffrey Ruth, Otto Lanz, Vincent Ziglioli

**Affiliations:** ^1^Virginia-Maryland College of Veterinary Medicine, Blacksburg, VA, USA

**Keywords:** whirl sign, cryptorchid, torsion, Sertoli cell tumor, computed tomography

## Abstract

An 11-year-old male German Shepherd dog presented for inappetence and weight loss. Physical examination and initial bloodwork revealed palpable abdominal masses, mild non-regenerative anemia, and thrombocytopenia. Survey radiography and abdominal ultrasonography confirmed the presence of bilateral abdominal masses and lymphadenopathy. Contrast-enhanced computed tomography (CT) was performed in order to further investigate the origin of the intraabdominal masses, confirming two enlarged cryptorchid testes, one of which had an associated CT “whirl sign.” Histopathology of the testes and lymph nodes revealed bilateral malignant Sertoli cell tumors and seminomas with lymph node metastasis of both neoplasms. The purpose of this case report is to discuss the benefits of CT in the diagnosis of cryptorchid testes and describe an additional organ that may display CT “whirl sign.”

## Case Presentation

An 11-year-old German Shepherd dog (40 kg) was evaluated at the veterinary teaching hospital for recent decreased appetite, mild weight loss, and an abdominal mass that was present for several months. The dog had no previous health problems prior to the episode and was up to date on vaccinations. No scrotum was present, and castration status was unknown. The most pertinent physical exam findings included diffuse muscle wasting, a large abdominal mass, and signs of feminization syndrome, namely prominent mammary glands and prostatomegaly, as revealed by transrectal palpation. Other signs of feminization syndrome, such as alopecia and skin hyperpigmentation, were not present. A complete blood count revealed mild non-regenerative anemia (HCT = 26.1%, range: 37.0–55.0%) with moderate thrombocytopenia (51 K/μL, range: 175–500 K/μL). Right lateral and ventrodorsal radiographs of both the thorax and abdomen were acquired, confirming multiple confluent soft tissue opaque peritoneal masses in the mid abdomen, caudoventral to the kidneys. The largest of these masses was a multi-lobular right-sided mass that spanned from the cranial to caudal abdomen and displaced abdominal viscera to the left. Peritoneal effusion was present, preventing complete assessment of the splenic silhouette. No nodular metastases were noted in the lungs. Differential diagnoses considered at the time of radiography included a soft tissue mass of splenic origin (primary splenic neoplasia, splenic torsion, hematoma) and/or marked lymphadenopathy. Disease of cryptorchid testicular origin was not initially considered due to the massive size of the lesion.

**Figure 1:**
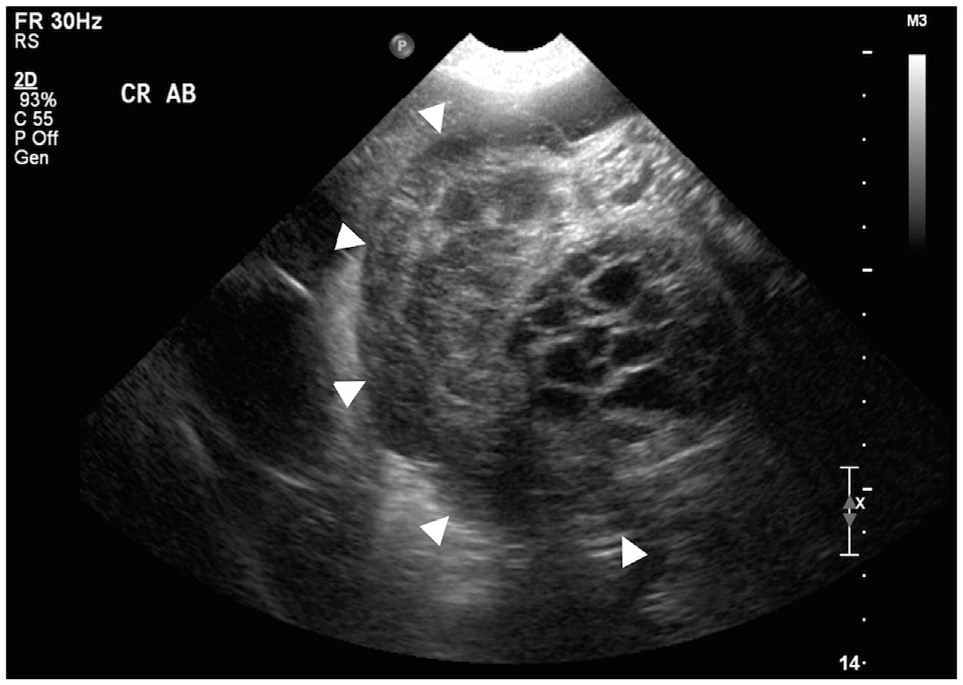
**Transverse sonographic view of the cranial abdomen displaying a transverse section of the largest mass (arrowheads surround the mass)**. Note the presence of intralesional cavitations.

Abdominal ultrasonography was performed with a broad bandwidth microconvex transducer (5–8 mHz) on a Phillips iU22 (Philips Medical Systems, Bothell, WA, USA). Examination revealed four separate masses and a moderate amount of echogenic fluid in the peritoneal cavity. The largest mass occupied the majority of the right abdominal cavity and had a cavitated appearance (Figure 1) with minimal perfusion on color flow Doppler. A second, rounded mass found in the left caudal abdomen also had a cavitated appearance similar to the largest mass. These bilateral lesions were distinctly separate from the kidneys, liver, and spleen, but the exact organ of origin could not be determined. The remaining two paired masses were presumed to be enlarged medial iliac lymph nodes based on their position lateral to the left and right external iliac arteries. The urinary bladder and prostate were not visualized, presumably due to caudal displacement from mass effect. The presence of intralesional cavitations and lymphadenopathy suggested neoplasia; however, the organ of origin was not identified.

**Figure 2:**
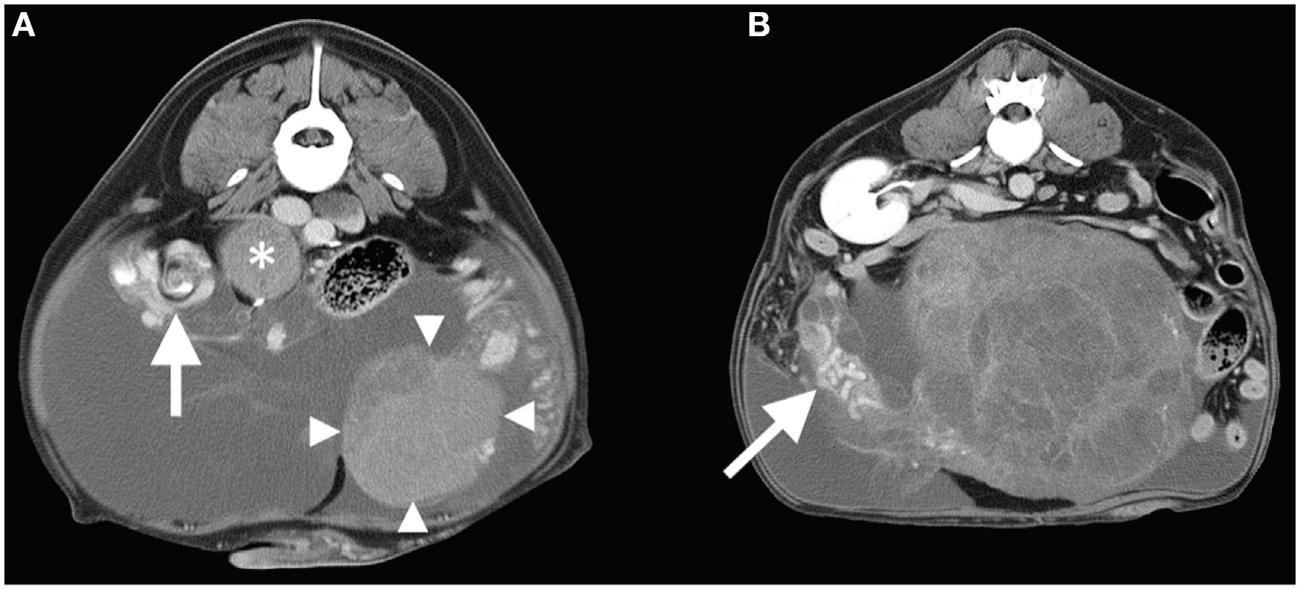
**(A)** Transverse post-contrast CT image at the level of the right testicular torsion (whirl-sign arrowed) and the left testicular mass (arrowheads). An adjacent enlarged lymph node is also labeled (*). The right side of the patient is oriented to the reader’s left. **(B)** Transverse post-contrast CT image at the level of the right kidney demonstrating the cavitated right testicular mass with enlarged tortuous vascular pedicle (arrow). Note that peritoneal effusion is evident in both images.

Therefore, thoracoabdominal computed tomography (CT) was performed to further investigate the lesion and to screen for pulmonary metastasis. Pre- and post-contrast images were acquired using a 16-slice helical CT scanner. The masses described in ultrasound examination were all distinctly visualized on the CT images. The two large cavitary masses each contained a cord-like vascular pedicle along the abaxial margin, containing both a venous plexus and arteries originating directly from the abdominal aorta between the caudal mesenteric and renal arteries. Based on the appearance of this associated vasculature, which was consistent with pampiniform plexuses and testicular arteries, respectively, the masses were determined to be testes. Both testicular masses exhibited heterogeneous contrast enhancement (Figures 2A,B). When compared with the left testicular mass, the right testicular mass exhibited a reduced degree of contrast enhancement suggesting hypoperfusion. Additionally, the vascular pedicle associated with the larger right testicular mass was focally arranged as a “whirl-like” structure comprised of spiraled striations of fat and heterogeneously contrast-enhancing soft tissue. The CT supported the ultrasound finding of medial iliac lymphadenopathy by detecting paired, irregularly shaped, soft tissue attenuating masses (up to 6.3-cm diameter) lateral to the external iliac arteries that exhibited heterogeneous contrast enhancement. Additional irregularly shaped, soft tissue attenuating structures with similar heterogeneous enhancement were detected in the expected locations of the sacral and right hypogastric lymph nodes and in the cranioventral mediastinum in the expected location of the right sternal lymph node. Within the subcutaneous fat adjacent to the external pudendal artery and vein, there were additional, rounded lymph nodes in the expected location of the inguinal lymph nodes. Further CT findings included moderate peritoneal effusion, symmetrical prostatomegaly, contrast-enhancing mammary gland tissue, and absence of pulmonary nodules.

Based on the CT findings, the working diagnosis was bilateral cryptorchidism with presumed malignant conversion, suspected partial torsion of the right testis, lymph node metastasis, and probable benign prostatic hyperplasia. The presence of mammary gland development suggested feminization secondary to a Sertoli cell tumor.

**Figure 3:**
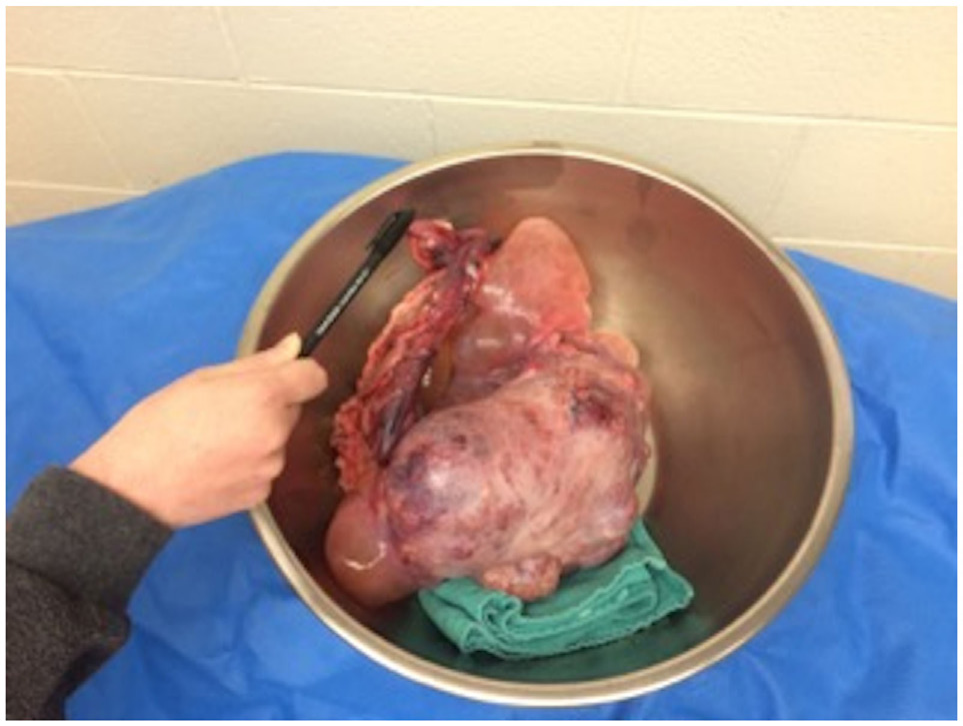
**Post surgical picture of the largest of the removed masses – the right testis**.

A celiotomy was performed immediately following the CT in order to remove the cryptorchid testes and enlarged lymph nodes. A mild–moderate amount of peritoneal fluid was removed. Bilateral testicular masses were confirmed. The right testis, approximately 25 cm, had a multilobulated, cystic appearance with 360° torsion of the spermatic cord (Figure 3). It was not determined if this was rotation of structures within the tunica vaginalis or extravaginal torsion of the spermatic cord. To a lesser degree, the left testis was also rotated on its pedicle. No thrombus was noted during surgery; therefore, it is possible that the rotation was not associated with impairment of the vasculature. Three abnormal sublumbar lymph nodes were also removed from the abdominal cavity. All other organs appeared normal upon further exploration of the abdominal cavity. On histopathology, both testes were found to have malignant Sertoli cell tumors and seminomas, both of which had metastasized to the lymph nodes. No thrombi were observed in either testicular pedicle. Treatment options with chemotherapy for residual metastatic disease were offered and declined by the owners.

## Background

Cryptorchidism is a well-documented developmental disease in dogs with a reported prevalence of up to 10% (1). Unilateral cryptorchidism is more often encountered than bilateral cryptorchid testes (2). Overall, cryptorchidism is a disease that can often be accompanied by an array of complicating factors, such as various forms of neoplasia and testicular torsion.

Cryptorchid dogs develop testicular neoplasms up to 13.6 times more frequently than normal dogs; with one study reporting that up to 5.7% of cryptorchid testes develop neoplasms (3, 4). Testicular neoplasms, in turn, are believed to expose the testes to a higher risk of torsion as a result of additional weight (5). Testicular torsion can be described as partial or complete, depending on the degree of rotation and occlusion of blood supply.

In a study of dogs seen at veterinary teaching hospitals across North America, the two most common neoplasms in cryptorchid testes were Sertoli cell tumors and seminomas (6). Both tumor types have been associated with feminization syndrome (7, 8). Feminization syndrome is caused by the over production of estrogens and is characterized by clinical signs that may include bilateral symmetric alopecia, atrophy of sebaceous glands, skin thinning, flaccidity of the prepuce, enlargement of mammary tissue, and prostate dystrophy (8).

Ultrasonography is a non-invasive and highly sensitive method for diagnosing cryptorchidism in dogs (9). The sonographic appearance of non-neoplastic and neoplastic canine cryptorchid testes is described. Non-neoplastic cryptorchid testes will be smaller, but have similar shape and sonographic pattern compared to descended testes. Neoplastic testes, whether descended or retained, are in general larger and have a mixed echogenic appearance (9, 10). Depending on the period of existing torsion, the sonographic appearance of the gonad may change, the acute torsion presenting as an enlarged testis with hypoechoic parenchyma and chronic torsions generally appearing small and hypoechoic (11, 12). In human medicine, the use of color flow Doppler to detect partial torsions has a high frequency of false-negatives due to the remaining presence of limited blood flow (13).

The limitations of abdominal ultrasonography have prompted consideration of abdominal CT. In veterinary literature, a previous example of a cryptorchid testis with a Sertoli cell tumor is described on CT as a well-defined soft tissue attenuating peritoneal mass and an associated pampiniform plexus (14). When compared with ultrasonography, abdominal CT provides a higher sensitivity in detecting non-neoplastic cryptorchid testes in people (15). This may also be true in large breed dogs, as CT has been reported to have greater sensitivity for detection of clinically relevant abdominal lesions when compared with ultrasound (16).

This case report provides an example of how CT can aid in the diagnosis of cryptorchidism and suspected testicular torsion. Although ultrasound was able to identify multiple abdominal masses and lymphadenopathy, CT verified that the masses were of testicular origin. Additionally, CT “whirl sign” was present, suggesting rotation of the spermatic cord or torsion.

## Discussion

Here, we present a case of bilaterally cryptorchid testes with malignant transformation and suspected torsion. Feminization signs included enlargement of mammary tissue and prostatomegaly. CT was ultimately utilized in order to make the diagnosis of testicular neoplasia with evidence of metastasis to lymph nodes. Signs of metastatic infiltration of lymph nodes on CT included irregular shape, enlarged size, and heterogeneous contrast enhancement (17).

The unique feature in this case report was the presence of CT “whirl sign” associated with the pedicle of the right testicular mass, indicating rotation of the testicular sustentatory structures with or without occlusion of vascular flow. Distal to the “whirl sign,” the right vascular pedicle was defined by a tortuous, contrast-enhanced artery and a tubular arrangement of non-enhanced lobulated soft tissue attenuating cavities, suggesting thrombosis or venous stasis within the pampiniform plexus. Suspicion of occlusion of right testicular venous return was present on gross examination during surgery. Incomplete obstruction of arterial blood flow, such as might happen in cases of partial torsion, could account for the CT finding of heterogeneous enhancement of the mass.

Despite these findings, occlusion of the vasculature within the spermatic cord may not have been present. A thrombus was not directly observed grossly or on histologic assessment, and acute abdominal pain associated with testicular congestion and necrosis was not present. Therefore, it is possible that the described appearance suggested venous stasis secondary to rotation of the vascular pedicle.

“Whirl sign” on CT is the result of the rotation of tissue and its associated vascular supply. Although the sensitivity and specificity of “whirl sign” as a sign of torsion has not been studied, it is used to help diagnose various forms of volvulus in abdominal organs. “Whirl sign” has been previously described in cases of intestinal, mesenteric, and splenic volvulus in dogs (18, 19). In the human literature, “whirl sign” has been described in cases of intestinal and mesenteric volvulus as well as in one case of gallbladder torsion (20, 21). In addition to its use as a radiographic indicator of volvulus, “whirl sign” can be used to guide clinical management of small bowel obstruction in human medicine and is a strong indicator that surgery is necessary to treat the obstruction (22).

To the best of the author’s knowledge, CT “whirl sign” associated with testicular torsion or spermatic cord rotation has not been previously reported in dogs. This case contributes to the current literature by adding an alternative organ for the presence of CT “whirl sign” as well as provide an additional imaging sign in cases of suspected testicular torsion. Abdominal CT should be considered when evaluating abdominal masses in large breed dogs, particularly if the organ of origin is unclear on other modalities.

## Concluding Remarks

This case report provides a description of cryptorchid testes with neoplastic conversion and suspected partial torsion, and presents an alternative organ for CT “whirl sign” in dogs. In addition, this report discusses an instance where radiography and ultrasonography were unable to determine the organ of origin of an abdominal mass; therefore, CT was performed for the diagnosis of bilateral cryptorchidism. High sensitivity and specificity as well as the ability to look for metastatic pulmonary nodules are among the many advantages of using CT for the investigation of abdominal lesions.

## Consent

Ethical approval and written consent from the owner were not needed for this report. The report was written retrospectively on a patient treated with standards of care at a veterinary teaching hospital. Patient care, including diagnosis and treatment, did not include methods intended for research.

## Author Contributions

SS contributed to writing the manuscript and literature review. JR contributed to writing the manuscript as well as interpreting and describing the imaging findings. OL contributed to writing the manuscript and performed the described surgery on the patient. VZ contributed to writing the manuscript and assisted with the described surgery on the patient.

## Conflict of Interest Statement

The authors declare that the research was conducted in the absence of any commercial or financial relationships that could be construed as a potential conflict of interest.
